# Deletion of OGG1 Results in a Differential Signature of Oxidized Purine Base Damage in mtDNA Regions

**DOI:** 10.3390/ijms20133302

**Published:** 2019-07-05

**Authors:** Guglielmina Chimienti, Vito Pesce, Flavio Fracasso, Francesco Russo, Nadja Cristhina de Souza-Pinto, Vilhelm A. Bohr, Angela Maria Serena Lezza

**Affiliations:** 1 Department of Biosciences, Biotechnologies and Biopharmaceutics, University of Bari Aldo Moro, Via Orabona 4, 70125 Bari, Italy; 2 Laboratory of Nutritional Pathophysiology, National Institute of Digestive Diseases–I.R.C.C.S. “Saverio de Bellis”, 70013 Castellana Grotte, Italy; 3 Department of Biochemistry, Chemistry Institute, University of São Paulo, São Paulo, SP 05508-000, Brasil; 4 Laboratory of Molecular Gerontology, National Institutes on Aging, National Institutes of Health, Baltimore, MD 21224, USA

**Keywords:** mtDNA repair, mtDNA content, OGG1 knockout (ko), NTH1 ko, 8-oxoG localization

## Abstract

Mitochondrial oxidative stress accumulates with aging and age-related diseases and induces alterations in mitochondrial DNA (mtDNA) content. Since mtDNA qualitative alterations are also associated with aging, repair of mtDNA damage is of great importance. The most relevant form of DNA repair in this context is base excision repair (BER), which removes oxidized bases such as 8-oxoguanine (8-oxoG) and thymine glycol through the action of the mitochondrial isoform of the specific 8-oxoG DNA glycosylase/apurinic or apyrimidinic (AP) lyase (OGG1) or the endonuclease III homolog (NTH1). Mouse strains lacking OGG1 (OGG1^−/−^) or NTH1 (NTH1^−/−^) were analyzed for mtDNA alterations. Interestingly, both knockout strains presented a significant increase in mtDNA content, suggestive of a compensatory mtDNA replication. The mtDNA “common deletion” was not detected in either knockout mouse strain, likely because of the young age of the mice. Formamidopyrimidine DNA glycosylase (Fpg)-sensitive sites accumulated in mtDNA from OGG1^−/−^ but not from NTH1^−/−^ mice. Interestingly, the D-loop region was most severely affected by the absence of OGG1, suggesting that this region may be a hotspot for oxidative damage. Thus, we speculate that mtDNA alterations may send a stress message to evoke cell changes through a retrograde mitochondrial–nucleus communication.

## 1. Introduction

Mitochondria are the powerhouses of the cell because of the large amount of ATP generated through oxidative phosphorylation. However, they are also fundamental hubs of cell metabolism due to their role in other essential processes including nutrient sensing, biosynthesis of precursors, redox regulation, disposal of metabolic waste, calcium regulation, and decision in cell fate [1]. Further, mitochondrial oxidative phosphorylation is the primary cellular source of reactive oxygen species (ROS). According to the mitohormesis theory, mitochondrial ROS generation may either contribute to increasing health and viability or induce damage in the physically close mitochondrial DNA (mtDNA), proteins, and lipids, as well as in other targets [2]. The prevalence of oxidant versus antioxidant species determines oxidative stress conditions that characterize aging, age-related diseases, and a large number of pathologies [3]. Oxidative stress is known to induce many cell responses, including the control of mitochondrial maintenance via the balance between biogenesis and mitophagy, with a direct effect on mtDNA content. Alterations in mtDNA content were reported in aging humans [4], mice [5], and rats [6], and in some pathological conditions including diabetic retinopathy [7] and celiac disease [8]. In addition to quantitative changes of mtDNA, qualitative damage accumulation including modified bases, abasic sites, single- and double-strand breaks (SSBs and DSBs), and large-size deletions was described with aging [9] and in various pathologies [10]. In particular, amongst the multiplicity of the reported mtDNA large-size deletions, there is one, flanked by direct repeats (DR1 and DR2), that eliminates a similar array of genes in a comparable size fragment in aged humans [11], rats [12], and mice [13], which is known as the “common deletion” because of its high incidence in tissues and subjects. Deleted mtDNA molecules usually constitute relevant fractions of the cellular mtDNA population, likely due to favored clonal expansion of the deleted genomes compared to the full-size molecules [14]. Accumulation of deleted molecules may then elicit detrimental consequences for mitochondrial and cell metabolism both in aging [4,15] and diseases [16]. Although the debate over the origin of such deletions is still ongoing and they are related to replication problems, other studies in vivo suggest that alterations in the DSB repair can also lead to deletions [17]. This drives attention to the repair mechanisms of mtDNA damage as a potential cause of the deletions. In mitochondria, the most relevant DNA repair pathway is base excision repair (BER) [18], which removes oxidized bases such as 8-oxoguanine (8-oxoG) through the action of the mitochondrial isoform of 8-oxoG DNA glycosylase/apurinic or apyrimidinic (AP) lyase (OGG1) [19], whereas oxidized pyrimidines including thymine glycol (5,6-dihydroxy-5,6-dihydrothymine) (Tg) are primarily repaired by the mitochondrial isoform of the endonuclease III homolog NTH1 [20]. The generation of mice defective in the *OGG1* gene [21] or the *NTH1* gene [22] provided us with models featuring the absence of specific base excision repair (BER) enzymes. The aim of the present study was to determine the roles of these two enzymes in mtDNA maintenance through the analysis of mtDNA and the “common deletion” contents, and the evaluation of the incidence of purine-specific lesions at determined regions.

## 2. Results

### 2.1. mtDNA Content and 3873-bp-Long Deletion

**Figure 1:**
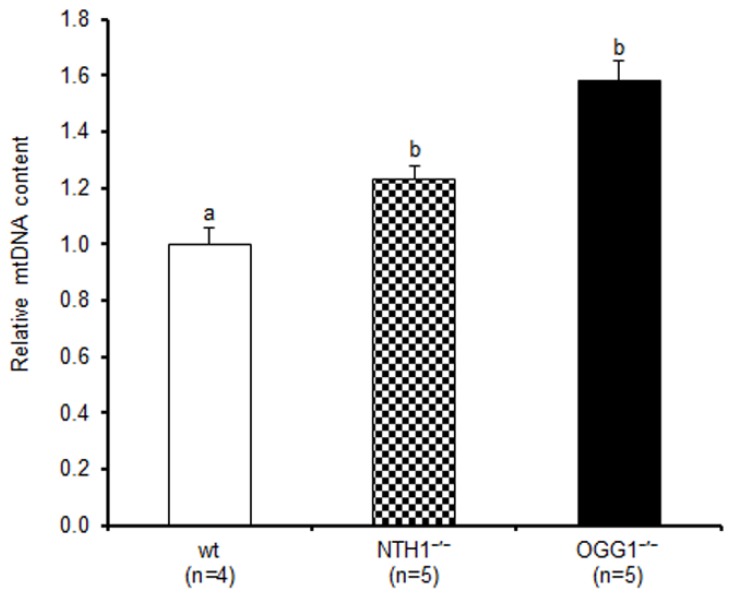
Relative mitochondrial DNA (mtDNA) content in liver from wild-type (wt), endonuclease III homolog knockout (NTH1^−/−^), and mitochondrial isoform of 8-oxoG DNA glycosylase/apurinic or apyrimidinic (AP) lyase knockout (OGG1^−/−^) mice. Bars represent the mean value and standard error of the mean (SEM) of the relative mtDNA content determined by qPCR of two independent experiments conducted in triplicates. Statistical difference was determined by the Kruskal–Wallis test with the Dunn’s multiple comparison test. Bars not sharing a common superscript letter differ significantly (*p* < 0.05, Dunn’s multiple comparison test); *n* = number of analyzed animals.

The relative mtDNA content was determined by qPCR in liver samples from four wild-type (wt), five NTH1^−/−^, and five OGG1^−/−^ mice, at four months of age (Figure 1). Significant increases in mtDNA content were observed in the knockout mice compared to wt: a 30% increase in the NTH1^−/−^ mice (*p* < 0.05, Dunn’s multiple comparison test) and a 58% increase in the OGG1^−/−^ mice (*p* < 0.001, Dunn’s multiple comparison test). No significant difference was found between NTH1^−/−^ and OGG1^−/−^ for relative mtDNA content.

**Figure 2:**
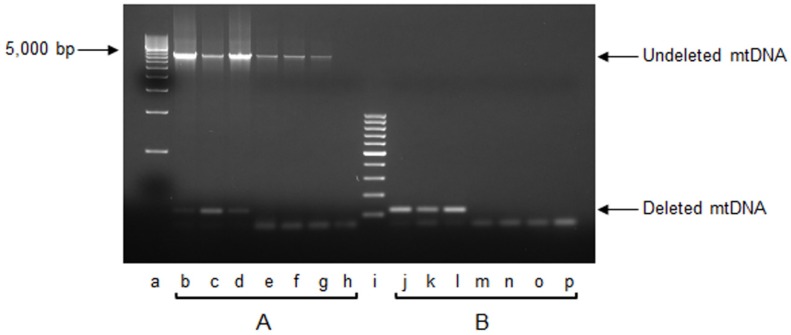
End-point PCR analysis of mouse mtDNA “common deletion”. Representative gel of end-point PCR amplification of total DNA isolated from liver of three 18-month-old wt mice, and three 4-month-old mice, belonging to wt, NTH1^−/−^, and OGG1^−/−^ strains, respectively. (**A**) extension step = 4 min; (**B**) extension step = 30 s. Amplicons (5-μL aliquots) were visualized by gel electrophoresis on ethidium bromide-stained 1.5% agarose gel. Lane loading was as follows: a = EZ load 500-bp molecular ruler (BioRad Laboratories Inc., Hercules, CA, USA); b, c, d = total DNA from each one of the three 18-month-old wt mice; e, f, g, = total DNA from one wt, one NTH1^−/−^, and one OGG1^−/−^ four-month-old mouse, respectively; h = blank with no DNA template; i, GeneRuler 100-bp DNA ladder (Thermo Fisher Scientific Inc, Waltham, MA, USA); j, k, l = total DNA from each one of three 18-month-old wt mice; m, n, o = total DNA from one wt, one NTH1^−/−^, and one OGG1^−/−^ four-month-old mouse, respectively; p = blank with no DNA template.

We investigated the presence of the mouse “common deletion”, a well-acknowledged marker of mitochondrial genome structural damage. This deletion [13] was initially reported to encompass 3,867 bp, but further studies, after the delivery of a new complete mouse mtDNA reference sequence (NC_005089.1), more accurately determined it to be 3873 bp long [23]. Using the qPCR technique, we did not detect the mtDNA “common deletion” in any of the assayed animals, independently of the genotype. Since this result might be due to the young age of the animals under investigation, we verified this negative result by applying the qPCR technique to total DNA samples obtained from the liver of three 18-month-old wt mice, taken as a plausible positive control. We did detect the deleted mtDNA product by qPCR in the 18-month-old mice (not shown). This was further confirmed by end-point PCR experiments performed with the same primers that were used for the qPCR. As shown in Figure 2, the presence of the amplicon derived from the shortened mtDNA molecules was found in the aged mice only, both when long (4 min; Figure 2, lanes b–d) and short (20 s; Figure 2, lanes j–l) extension times were applied. When the long extension time was used, it was possible to also obtain the amplicon derived from undeleted genomes in all assayed animals (Figure 2, lanes b–g). This observation confirmed the above results, showing the absence of the “common deletion” in the younger mice tested. 

### 2.2. Analysis of Oxidized Purines

**Figure 3:**
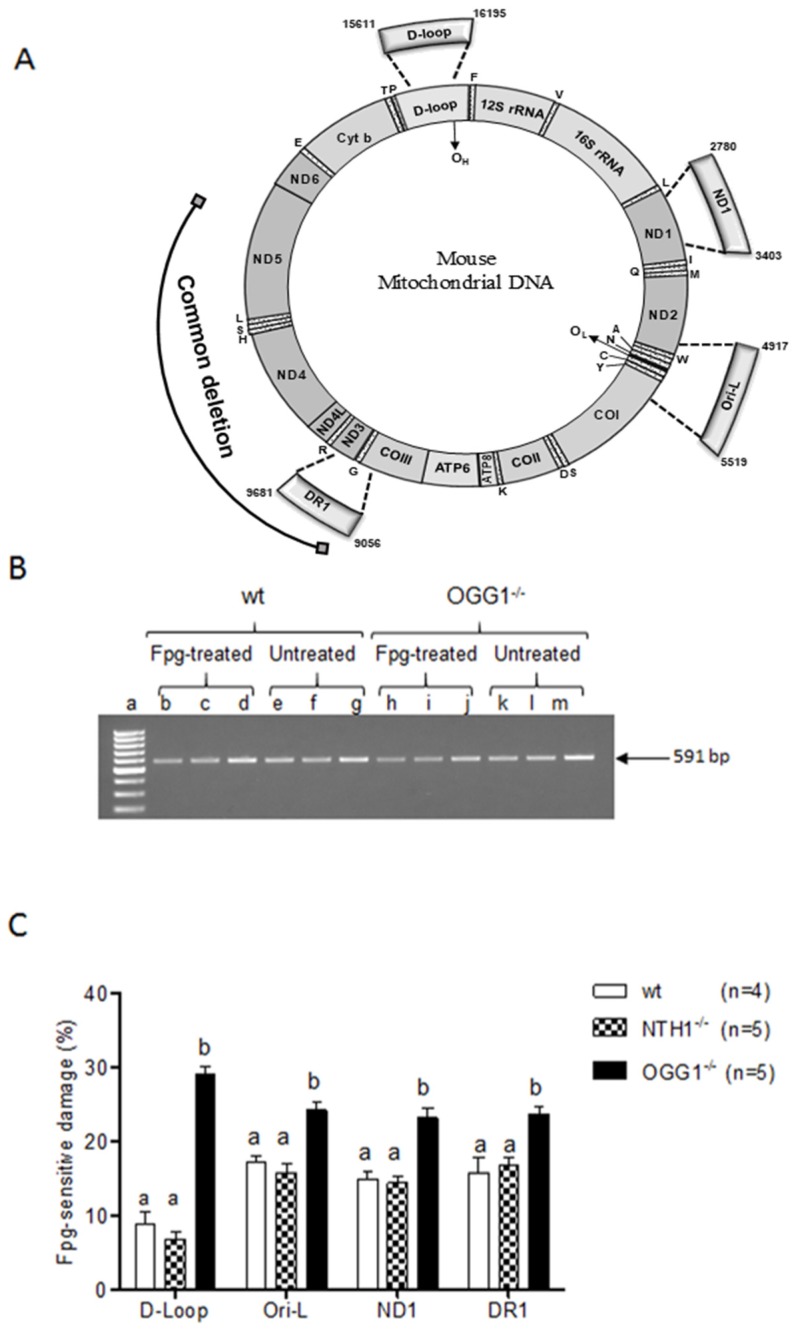
Analysis of purine-specific mtDNA damage at the d-loop, Ori-l, ND1, and direct repeat 1 (DR1) regions in liver from wt, NTH1^−/−^, and OGG1^−/−^ mice. (**A**) Map of mouse mtDNA. The thick arches extending out of the circle represent the four amplified regions tested for formamidopyrimidine DNA glycosylase (Fpg) sensitivity delimited, respectively, by the primer pairs listed in Table 2, and corresponding to the indicated nucleotides. Numbering is according to National Center for Biotechnology Information (NCBI) accession number NC_005089.1. The outmost arch represents the 3.8-kb deletion, delimited by direct repeat 1 (DR1) and direct repeat 2 (DR2) represented by two full squares. (**B**) representative gel analysis of amplicons from Fpg-treated and untreated total DNA from one wt and one OGG1^−/−^ mouse (the target was the d-loop region). An aliquot (5 μL) of each PCR reaction was loaded onto a 1.5% agarose ethidium bromide-stained gel and analyzed for band intensities. Lane loading was as follows: a = GeneRuler 100-bp DNA ladder (Thermo Fisher Scientific Inc, Waltham, MA, USA); b, c, e, f, h, i, k, and l = 5 ng of total DNA as template; d, g, j, and m = 7.5 ng of total DNA as template. (**C**) Bars represent the mean and SEM of the ratio between Fpg-treated and untreated band intensities, expressed as the percentage of the complement to 100 to improve the graphical evaluation. Statistical difference determined by the Kruskal–Wallis test with Dunn’s multiple comparison test. Bars not sharing a common superscript letter differ significantly (*p* < 0.05).

Previous studies conducted in the same groups of mice revealed increased amounts of 8-oxoG in the OGG1^−/−^ strain compared to wt and NTH1^−/−^ strains [24,25]. Thus, it was of interest to verify if the oxidized base was evenly distributed along the mtDNA molecule or not. Therefore, four mtDNA regions were screened for the incidence of 8-oxoG. The first two regions encompassed the origins of replication of mtDNA (namely the d-loop and Ori-l, respectively), the third region included the DR1 repeat of the “common deletion”, and the fourth was in the *ND1* gene, which constituted a control coding region not involved, so far, in genomic instability events (Figure 3A). The presence of oxidized purines, among which 8-oxoG is likely to be the predominant, was detected using the oxidized purine-sensitive enzyme formamidopyrimidine DNA glycosylase (Fpg), and the incidence of the Fpg-sensitive sites was calculated as the ratio between the amount of PCR-amplifiable template from the Fpg-treated and from the untreated DNA counterpart, as a percentage (Figure 3B). Results were shown as the complement relative to 100. The incidence of Fpg-sensitive sites was significantly higher in the OGG1^−/−^ than in wt animals in all four assayed regions (*p* < 0.01: Ori-l and ND1; *p* < 0.001: d-loop and DR1, Dunn’s multiple comparison test), confirming the previous quantitative findings [24,25]. The incidence in the OGG1^−/−^ mice was also higher compared to that in NTH1^−/−^ ones, with a significant *p* < 0.001 in all four analyzed regions (Dunn’s multiple comparison test). The absence of significant differences in the incidence of Fpg-sensitive sites between NTH1^−/−^ and wt mice in each assayed region further confirmed the specificity of the DNA damage derived from the absence of the mitochondrial OGG1 enzyme (Figure 3C). 

We then compared the incidence of Fpg-sensitive sites in each strain among the four regions (Table 1). When the wt and NTH1^−/−^ strains were considered, the incidence of Fpg-sensitive sites was lower at the d-loop than in the other regions. These findings suggest that functional OGG1 appears to be more prone to repairing this critical functional region than the other ones. The Dunn’s *post hoc* test assessed the significance of the differences for Ori-l (*p* < 0.01) and DR1 (*p* < 0.05) vs. d-loop in wt mice, and for ND1 (*p* < 0.01) and for Ori-l and DR1 (*p* < 0.001, respectively) vs. d-Loop in NTH1^−/−^ mice. On the other hand, when the incidence of Fpg-sensitive sites was compared among the four analyzed regions in OGG1^−/−^ mice, the d-loop presented the highest value, significantly different from those of the other regions (*p* < 0.001, Dunn’s multiple comparison test), allowing its identification as a hotspot for oxidized base formation in the absence of an adequate repair system. 

## 3. Discussion

### 3.1. Effects on mtDNA Content and Integrity

The present results broaden our knowledge about the relevance of two specific enzymes active in the BER processing of mtDNA and shed new light on the cellular effects induced by their genetic ablation, thus helping unveil the gene-specific contribution to pathways leading to conditions common in aging and age-related diseases. The mouse strains used here were defective for OGG1 or NTH1 and were both viable and healthy into adulthood. In particular, the absence of an increased frequency of tumors in the OGG1^−/−^ mouse, in spite of the strong mutagenicity of the accumulated 8-oxoG, supports the existence of an alternative pathway for the repair of the oxidized base in the nuclear genome [21]. Similarly, compensatory pathways for the removal of Tg and other oxidized pyrimidines were implicated, because of the experimental results, in the NTH1^−/−^ mouse [22]. The consequences of lacking each of the two DNA glycosylases were severe for the mitochondrial DNA repair [19,20]. In particular, the OGG1^−/−^ mouse presented a 20-fold increased content of 8-oxoG in mtDNA compared to the wt counterpart, and its mitochondrial extract showed a total lack of incision activity for this lesion [19]. Similarly, no incision activity for Tg was detected in the mitochondrial extract from the NTH1^−/−^ mouse [20]. Therefore, the common trait of both knockout strains was the increased load of specific mtDNA lesions that could not be repaired and that, according to our present findings, unexpectedly evoked a significant increase in mtDNA content (Figure 1). The increase was higher in the OGG1^−/−^ mice (+58%) than in the NTH1^−/−^ animals (+30%), suggesting that the accumulation of each specific DNA lesion induced a different extent of compensatory mtDNA replication, probably related to the biological relevance of the respective lesion. Previous findings obtained in these same cohorts of knockout mice exclude an increase in the corresponding number of mitochondria per liver cell in comparison to wt animals. Effectively, the determination, by western blot experiments, of the amount of the mitochondrial cytochrome oxidase subunit IV revealed no differences in liver mitochondrial content among wt, NTH1^−/−^, and OGG1^−/−^ mouse strains [20]. Furthermore, the determination of citrate synthase activity in OGG1^−/−^ liver also clearly indicated no change in mitochondrial mass with respect to wt mice [26]. Therefore, the present results highlight a real increase in mtDNA content in liver from both knockout strains. Such an increase in mtDNA content points to a whole-cell response to damage accumulation sensed at the mitochondrial level and communicated to the nucleus through a retrograde response. Recent reports highlighted how mtDNA damage or dysfunctional oxidative phosphorylation may impinge upon nuclear DNA expression through increased ROS signaling, Ca^2+^ efflux, or decreased ATP availability [27]. Such pathways might lead to an increased mtDNA content in an effort to make up for the reduced mitochondrial bioenergetics. An increased mtDNA content was described previously in various human pathologies featuring mild oxidative stress such as skeletal muscle aging [4], patients suffering from type 2 diabetes [28], patients with diabetic retinopathy [7], or patients with celiac disease [8]. 

Another well-known marker of mitochondrial dysfunction, related to an oxidative stress situation, is the presence of the large-size so-called “common deletion”, described in various tissues from aging organisms [11,12,13] and patients [29]. In our search for the 3873-bp-long mouse “common deletion”, as shown in Figure 2, we could find no evidence of the “common deletion” in four-month-old mice irrespective of strain. We suggest that this negative result might be due to the young age of the animals, since when the analysis was performed on DNA obtained from livers from three aged (18-month-old) wt mice, the deletion was detected, thus confirming previous results from ours and other groups [4,9,13]. Indeed, recent evidence suggests that mtDNA large-size deletions might arise from errors in SSB [30] or DSB [17] repair. Indications consistent with this idea might be inferred from a very recent study demonstrating the relevance of the γ-DNA polymerase, which is the major DNA polymerase involved in the repair of mtDNA, in preventing the formation of deletions through removal of linear DNA fragments originated from unrepaired DSBs [31]. The absence of the “common deletion” in both knockout groups, each lacking a specific component of the BER system, indirectly supports the above-reported findings [17,30,31].

### 3.2. Oxidized Purine Damage at Specific mtDNA Regions

Another aim of this study was to determine whether the 8-oxoG accumulated in the OGG1^−/−^ mtDNA [19] was homogeneously present along the molecule or localized at damage hotspots. The Fpg assay revealed that the d-loop region was the most severely affected by the absence of the DNA glycosylase, leading to a significant accumulation of oxidized purines, thus confirming other reports that identified this region as a hotspot extremely sensitive to oxidative damage [9,32]. 

Interestingly, in the presence of functional OGG1 (wt and NTH1^−/−^ strains), the d-loop region appeared to be protected from the accumulation of oxidized purines compared to the other regions in mtDNA. As a plausible explanation for this finding, we suggest that the normal DNA repair activity of the OGG1 enzyme might be reinforced by other factors at this crucial functional region to reduce the mutagenic potential of 8-oxoG and other oxidized purines. 

Effectively, the d-loop portion that we examined through the Fpg assay included conserved sequence block 1 (CSB1) and 2 (CSB2) [33], where mitochondrial transcription factor A (TFAM) binding sites were identified in rat [34] and human mtDNA [35]. Given the large similarity between rat and mouse mitochondrial genomes, we speculate that TFAM might usually bind to the analyzed mouse d-loop segment to further shield it from oxidative damage. The functional relevance of the region might be the reason for such extra-protection because it includes the origin of replication for the H strand (O_H_), and this is likely important for the maintenance of mtDNA. Data obtained by Pastuck et al. (2016) [32] in pulmonary artery endothelial cells challenged with mild oxidative stress in the form of hypoxia support our results. These authors also found an accumulation of 8-oxoG in the d-loop region and an increased mtDNA content [32]. Thus, we suggest that the accumulation of oxidized purines at the d-loop or some other oxidative damage-related signaling in the OGG1^−/−^ strain might induce a retrograde communication leading to the increased mtDNA content. Actually, a growing number of studies demonstrate that, through different kinds of signaling (energetic stress, Ca^2+^-dependent stress, ROS-dependent stress, and others), retrograde communication induces a nuclear expression response resulting in metabolic reprogramming or stress defense [27,36,37,38]. An indirect confirmation of the relevance of the absence of OGG1 for whole-cell metabolism derives from recent reports demonstrating marked tissue-specific metabolic changes mediated by alterations of mitochondrial function in the OGG1^−/−^ mouse [39,40,41,42]. A similar response, induced by a specific retrograde communication, might also occur in the NTH1^−/−^ mouse evoking the increase in mtDNA content. The present results highlight the need to characterize further the mtDNA changes sequential to the absence of specific members of the BER system [43] because of the related possible retrograde signaling, able to induce metabolic alterations [44] common to aging and age-related pathologies.

## 4. Materials and Methods

### 4.1. Mouse Samples

Knockout mice, with targeted deletions of the *OGG1* or *NTH1* genes, were kindly provided by Arne Klungland (University of Oslo, Norway) (OGG1^−/−^) [21] and Rhod Elder (Paterson Cancer Institute, Manchester, UK) (NTH1^−/−^) [22] and bred at the Gerontology Research Center (GRC) Animal Facility (Baltimore, MD, USA). Wild-type littermates (wt) were used as controls. For each experimental group, four-month-old mice were sacrificed by cervical dislocation, and the livers were immediately removed, washed free of blood with isolation buffer, and processed as described below. 

### 4.2. Isolation of Liver Genomic DNA

Total genomic DNA was isolated from the livers of four-month-old mice following a modification of the salting-out method already described [25]. 

### 4.3. Measurement of mtDNA Content and Detection of mtDNA 3873-bp-Long Deletion

Quantitative real-time polymerase chain reaction (qPCR) was used to measure mtDNA content (mtDNA primer set; Table 2) relative to nuclear DNA (β-actin primer set; Table 2), and the content of the mtDNA 3873-bp-long deletion (3.8 Del primer set; Table 2) normalized to mtDNA (mtDNA primer set). Reactions to measure the mouse mtDNA relative content were performed via SYBR Green chemistry as previously reported in a rat study [45].

The 3.8 Del primer set was also used to perform end-point PCR using total DNA from three 18-month-old mice as the template. PCR conditions were Dream Taq PCR 1× Master Mix (Thermo Fisher Scientific Inc, Waltham, MA, USA), 0.5 μM forward and reverse primers, and DNA template (20 ng) in 20 μL of final volume. PCR parameters were initial denaturation at 95 °C for 10 min, followed by 40 cycles of 30 s at 95 °C, 20 s at 60 °C, 20 s or 4 min at 72 °C, and 5 min of final extension at 72 °C. The amplicons (5-μL aliquots) were visualized by gel electrophoresis through a 1.5% agarose gel.

### 4.4. Modified Purines Analysis

Oxidized purines were detected using formamidopyrimidine DNA glycosylase (Fpg) (New England Biolabs, Beverly, MA, USA) digestion of total DNA [32].

The SSBs introduced by the selective cleavage by Fpg at sites of oxidized purines blocked the amplification reaction. The PCR amplification of the ND1, Ori-l, DR1, and D-loop mtDNA regions was conducted using the respective long primer sets (Table 2). The reaction mix (total volume of 20 μL) consisted of DreamTaq Green PCR 1× Master Mix (Thermo Fisher Scientific Inc, Waltham, MA, USA), 0.5 μM each forward and reverse primer, and template DNA (5 and 7.5 ng of Fpg-treated or untreated total DNA, respectively). The cycling conditions included a pre-incubation step of 10 min at 95 °C followed by 18 cycles of 15 s at 95 °C, 15 s at 60 °C, and 1 min at 72 °C. An aliquot (5 μL) of each PCR amplification was loaded on 1.3% agarose gel. Ethidium bromide-stained bands were visualized using the Gel Doc XR documentation system (BioRad Laboratories Inc., Hercules, CA, USA). Image Lab Software (BioRad Laboratories Inc., Hercules, CA, USA) was used to analyze band intensity. The value of the ratio between Fpg-treated and untreated band intensities was expressed as the percentage of the complement to 100 to improve the graphical evaluation.

### 4.5. Statistical Analysis

Data are presented as mean and standard error of the mean (SEM). Data were analyzed by Kruskal–Wallis test with Dunn’s multiple comparison test. All differences were considered significant at a 5% level. A specific statistical package was used (Stata Corp., 2005. Stata Statistical Software: Release. College Station, TX, USA).

## 5. Conclusions

From our results and recent literature data, it appears essential to analyze in detail the mtDNA changes induced by the absence of OGG1 and other DNA repair enzymes, as mtDNA alterations might represent the stress message sent to evoke, through a retrograde mitochondria–nucleus communication, changes in cellular and whole-organism metabolism. 

## Figures and Tables

**Table 1:** Formamidopyrimidine DNA glycosylase (Fpg)-sensitive damage (%) at specific mitochondrial DNA (mtDNA) regions in the three mouse strains. DR1—direct repeat 1; wt—wild type; NTH1^−/−^—endonuclease III homolog knockout; OGG1^−/−^—mitochondrial isoform of 8-oxoG DNA glycosylase/apurinic or apyrimidinic (AP) lyase knockout.

	mtDNA Region			
Strain	d-loop	Ori-l	ND1	DR1
wt	8.81 ± 1.64 ^a^	17.16 ± 0.94 ^b^	14.93 ± 1.12 ^ab^	15.73 ± 2.08 ^b^
NTH1^−/−^	6.79 ± 1.05 ^a^	15.77 ± 1.25 ^b^	14.49 ± 0.87 ^b^	16.77 ± 1.00 ^b^
OGG1^−/−^	29.10 ± 1.04 ^a^	24.27 ± 1.17 ^b^	23.20 ± 1.36 ^b^	23.79 ± 0.85 ^b^

Data are the mean ± standard error of the mean (SEM) of the ratio between Fpg-treated and untreated band intensities, expressed as the percentage of the complement to 100. Statistical difference determined by the Kruskal–Wallis test with Dunn’s multiple comparison test. Mean values not sharing a common superscript letter differ significantly (*p* < 0.05).

**Table 2:** Oligonucleotide primer sequences.

Primer Set	Forward Primer (5’–3’)	Reverse Primer (5’–3’)	(nps)	(nps)
mtDNA	AATCTACCATCCTCCGTGAAACC	GCCCGGAGCGAGAAGAG	15,687–15,709	15,748–15,732
β-actin	AGCCATGTACGTAGCCATCCA	TCTCCGGAGTCCATCACAATG	499–519	579–559
3.8 Del	AGCCTTATAGAAGGTAAACGAAACC	ACGCGGTTTTGTTATTGTTACG	9054–9078	13,050–13,029
ND1 long	CGTCCCCATTCTAATCGCCA	AAGGCTACGGCAAATTCAAG	2780–2799	3403–3384
Ori-l long	ATAACCCTACCCCTAGCCCC	ACAAAAGCATGGGCAGTTACG	4916–4935	5518–5498
DR1 long	GCCTAATAGAAGGTAAACGAAACC	CTATTCCTGCTCAGGCTCCA	9055–9078	9680–9656
d-loop long	GTGTTATCTGACATACACCATACAG	TGGGAACTACTAGAATTGATCAGGA	15,611–15,635	16,201–16,177

Numbering is according to National Center for Biotechnology Information (NCBI) accession number NC_005089.1 (*Mus musculus* mitochondrion, complete genome), except for the β-actin primer set which is according to GenBank™ accession number NM_007393 (*Mus musculus*, β-actin messenger RNA (mRNA)). nps: nucleotide positions.

## Abbreviations

OGG1	8-oxoG DNA glycosylase/AP lyase
mtDNA	mitochondrial DNA
ROS	reactive oxygen species
SSBs and DSBs	single- and double-strand breaks
BER	base excision repair
NTH1	endonuclease III-like protein 1
Fpg	formamidopyrimidine DNA glycosylase
